# Isolation and complete genome sequence of the thermophilic *Geobacillus* sp. 12AMOR1 from an Arctic deep-sea hydrothermal vent site

**DOI:** 10.1186/s40793-016-0137-y

**Published:** 2016-02-24

**Authors:** Juliane Wissuwa, Runar Stokke, Anita-Elin Fedøy, Kjersti Lian, Arne Oskar Smalås, Ida Helene Steen

**Affiliations:** Centre for Geobiology, University of Bergen, N-5020 Bergen, Norway; Department of Biology, University of Bergen, N-5020 Bergen, Norway; The Norwegian Structural Biology Center (NorStruct), Department of Chemistry, UIT - The Arctic University of Norway, 9037 Tromsø, Norway

**Keywords:** Thermophile, *Geobacillus*, Enzymes, Bioprospecting

## Abstract

Members of the genus *Geobacillus* have been isolated from a wide variety of habitats worldwide and are the subject for targeted enzyme utilization in various industrial applications. Here we report the isolation and complete genome sequence of the thermophilic starch-degrading *Geobacillus* sp. 12AMOR1. The strain 12AMOR1 was isolated from deep-sea hot sediment at the Jan Mayen hydrothermal Vent Site. *Geobacillus* sp. 12AMOR1 consists of a 3,410,035 bp circular chromosome and a 32,689 bp plasmid with a G + C content of 52 % and 47 %, respectively. The genome comprises 3323 protein-coding genes, 88 tRNA species and 10 rRNA operons. The isolate grows on a suite of sugars, complex polysaccharides and proteinous carbon sources. Accordingly, a versatility of genes encoding carbohydrate-active enzymes (CAZy) and peptidases were identified in the genome. Expression, purification and characterization of an enzyme of the glycoside hydrolase family 13 revealed a starch-degrading capacity and high thermal stability with a melting temperature of 76.4 °C. Altogether, the data obtained point to a new isolate from a marine hydrothermal vent with a large bioprospecting potential.

**Electronic supplementary material:**

The online version of this article (doi:10.1186/s40793-016-0137-y) contains supplementary material, which is available to authorized users.

## Introduction

In 2001 the genus *Geobacillus* was proposed by Nazina *et al.* [1] to distinguish it from the genus *Bacillus*. Bacteria of the genus *Geobacillus* have been isolated from diverse marine and terrestrial habitats such as oil wells [2], cool soils like from Bolivian Andes [3], sediments from Mariana Trench [4] and deep sea hydrothermal vents [5]. Surprisingly, these thermophiles can be isolated from cold environments from different geographical regions in such large quantities that it speaks against a “contamination” from hot environments, which have been described as paradox [6]. The influence of direct heating action of the sun upon the upper soil layers and heat development due to putrefactive and fermentative processes of mesophiles could give an explanation for their abundance [7, 8]. To our knowledge, *Geobacillus* has not been isolated from an Arctic marine habitat. As of June 2015, 37 *Geobacillus* genomes have been deposited in GenBank. Due to the development of next generation sequencing the number of new sequenced genomes (17) has been almost doubled in the last one and a half years. Of all *Geobacillus* genomes, 13 have been described as complete, whilst the other 24 genomes have been deposited as drafts. The genus exhibits a broad repertoire of hydrolytic and modifying enzymes and is therefore a valuable resource for biocatalysts involved in biotechnological processes with accelerated temperatures [9, 10]. The application of thermophilic microorganisms or enzymes in biotechnology gives advantage in enhancing biomass conversion in a variety of biotechnical applications; it minimizes contamination and can reduce the process costs [11]. Diverse *Geobacillus* strains comprise an arsenal of complex polysaccharide degrading enzymes such as for lignocellulose [12]. Other *Geobacillus* strains are able to degrade a broad range of alkanes [13, 14]. Up to now a multiplicity of patents derived from the genus comprises restriction nucleases, DNA polymerases, α-amylases, xylanase, catalase, lipases and neutral protease among others (EP 2392651, US2011020897, EP2623591, US2012309063, KR100807275 [15, 16]). The glycoside hydrolase group 13 (GH13) α-amylases are well studied enzymes which have a broad biotechnological application, for example for bioethanol production, food processing or in textile and paper industry [17]. Due to the broad application of α-amylases there is a focus of interest to identify novel α-amylases for new and improved applications in biotechnology. In addition to functional screening for enzyme activity, genome investigation is a valuable tool to identify potential biocatalysts. Here we present the isolation and metabolic features of *Geobacillus* sp. 12AMOR1 (DSM 100439) together with the description of the complete genome and its annotation.

## Organism information

### Classification and features

*Geobacillus* sp. strain 12AMOR1 was isolated from a 90°C hot deep-sea sediment sample collected in July of 2012 from the Arctic Jan Mayen Vent Field (JMVF). The sample was collected using a shovel box connected to a Remote Operating Vehicle (ROV) at a water depth of 470 m. The detailed description of the JMVF site is described elsewhere [18, 19].

The bacterium was isolated at 60°C on *Archaeoglobus* medium agar plates [20] pH 6.3 containing 1 % Starch (Sigma Aldrich) at the attempt to screen for starch degraders. Genomic DNA of isolates was extracted using FastDNA® Spin Kit for Soil (MP). The partial 16S rRNA gene was amplified by PCR using Hot Star Plus (QIAGEN) and following universal primers B8f (5’ AGAGTTTGATCCTGGCTCAG) [21] and Un1391r (5’ GACGGGCGGTGWGTRCA) [22]. The preliminary partial 16S rRNA gene fragment of strain 12AMOR1 has been analyzed using the megablast algorithm in the standalone blastn [23] against 16S ribosomal RNA (*Bacteria* and *Archaea* database). The partial 16S rRNA gene shared 98 % sequence identity with the strains *G. stearothermophilus*DSM 22^T^ (NR_114762.1) and R-35646 (NR_116987.1), as well as to other *Geobacillus* species: *Geobacillus subterraneus* strain 34^T^ (NR_025109.1), *Geobacillus**zalihae* strain NBRC 101842^T^ (NR_114014.1), *Geobacillus thermoleovorans* strain BGSC 96A1^T^ (ref|NR_115286.1), *Geobacillus thermocatenulatus* strain BGSC 93A1^T^ (NR_043020.1), *Geobacillus vulcani* strain 3S-1^T^ (NR_025426.1) and *Geobacillus kaustophilus* strain BGSC 90A1^T^ (NR_115285.1) (Additional file 1). The genome of *Geobacillus* sp. 12AMOR1 encoded 10 genes for 16S rRNA whereby blastn analysis [23] revealed small differences in top hits towards multiple *Geobacillus* strains. The 16S rRNA gene GARCT_01776 was identical to the partial sequence obtained by PCR mentioned above, and thus, the whole 16S rRNA gene GARCT_01776 was used for the phylogenetic analysis.

**Fig. 1:**
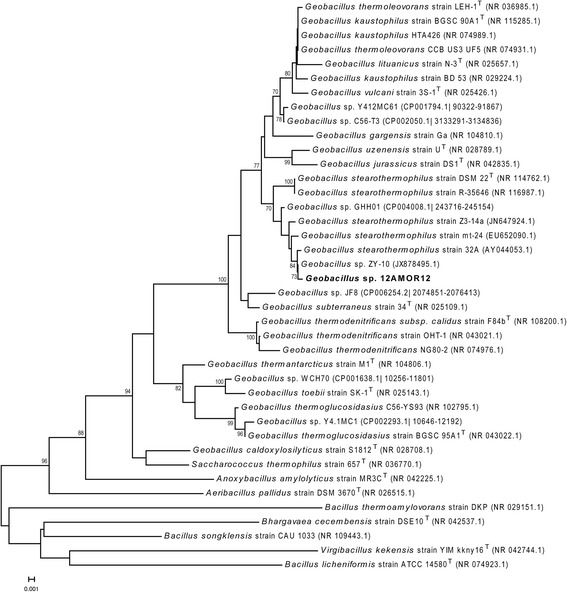
Phylogenetic tree showing the position of *Geobacillus* strain 12AMOR1 relative to the other strains of *Geobacillus* based on 16S rRNA. The Neighbor-Joining tree was built from 1374 aligned positions of the 16S rRNA gene sequences and derived based on the Tamura 3-parameter as preferred model and gamma distribution (shape parameter = 1) for modeling rates variation among sites using MEGA6. Bootstrap values above 70, expressed as percentage of 1000 replicates, are shown at branch points. Bar: 0.01 substitutions per nucleotide position. *Bacillus songklensis* strain CAU 1033 (NR_109443.1), *Bhargavaea cecembensis* strain DSE10 (NR_042537.1), *Bacillus licheniformis* strain ATCC 14580^T^ (NR_074923.1), *Virgibacillus kekensis* strain YIM kkny16^T^ (NR_042744.1) and *Bacillus thermoamylovorans* strain DKP (NR_029151.1) was used as outgroup

**Fig. 2:**
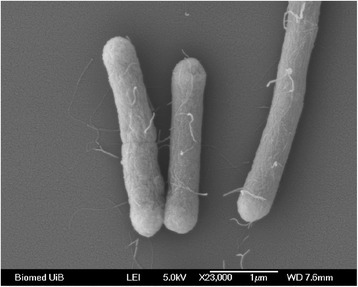
Scanning electron microscopy of *Geobacillus* sp. strain 12AMOR1

A phylogenetic tree was constructed from aligning the 16S rRNA gene GARCT_01776 with 16S rRNA genes from selected strains and species from the same genus using MUSCLE [24, 25] and Neighbor-Joining algorithm incorporated in MEGA 6.06 [26]. The 16S rRNA from *Geobacillus* sp. 12AMOR1 grouped together with *Geobacillus* sp. ZY-10 and *G. stearothermophilus* strain 32A, Z3-14a and mt-24 (Fig. 1). Interestingly, within the sub-cluster of *G. stearothermophilus*, the isolate 12AMOR1 and herein before mentioned strains were grouped apart from the type strain *G. stearothermophilus*DSM 22^T^*.* To further evaluate how closely related the new isolate was to existing species of *Geobacillus**,* a digital DNA-DNA hybridization (DDH) [27] was performed using the complete genomes of 13 *Geobacillus* species listed in Additional file 2. DDH estimations below 70 % suggested that *Geobacillus* sp. 12AMOR1 belonged to a new species. The level of relatedness by DDH estimations using formula 2 (identities/HSP length) ranged from 21.5 to 41.5 % between the isolate and different *Geobacillus* species. *Geobacillus* sp. 12AMOR1 is a Gram-positive [28], spore-forming, motile, facultative anaerobic rod. The cells are in average 0.5–0.7 μm in width and between 1.8 and 4.5 μm long. In addition, cells forming long filamentous chains were observed by microscopy. The cells were peritrichous flagellated (Fig. 2) consistent with previously observation of *Geobacilli* [1, 29]. Terminal ellipse shaped spores was observed.

The isolate was able to grow in a temperature range of 40 to 70 °C and pH of 5.5 to 9.0, with a temperature optimum of 60 °C and a broad pH optimum between 6.5 and 8.0. Growth was observed in concentrations ranging between 0 and 5 % NaCl. Besides aerobic growth, *Geobacillus* sp. 12AMOR1 was able to grow on yeast extract in anaerobic NRB medium containing nitrate [30].

Besides the utilization of starch, *Geobacillus* 12AMOR1 was able to grow on complex polysaccharides such as xylan, chitin and α-cellulose (Table 1). Fast growth was accomplished by cultivating the isolate on yeast extract and gelatin. In addition, the isolate utilizes lactose, galactose and organic acids such as lactate and acetate. No growth was observed using pectine, xylose, tween20 and tween80 as carbon source. *Geobacillus* sp. 12AMOR1 degrades DNA supplemented in agar (Fig. 4d).

**Table 1:** Classification and general feature of *Geobacillus* sp. strain 12AMOR1 according to the MIGS recommendations

MIGS ID	Property	Term	Evidence code
	Classification	Domain *Bacteria*	TAS [55]
		Phylum *Firmicutes*	TAS [56, 57]
		Class *Bacilli*	TAS [58, 59]
		Order *Bacillales*	TAS [60, 61]
		Family *Bacillaceae*	TAS [61, 62]
		Genus *Geobacillus*	TAS [1, 7, 29]
		Species *Geobacillus* sp.	IDA
		Strain 12AMOR1	IDA
	Gram stain	Positive	IDA
	Cell shape	Rod	IDA
	Motility	Motile	IDA
	Sporulation	Spore forming	IDA
	Temperature range	40-70 °C	IDA
	Optimum Temperature	60 °C	IDA
	pH range, optimum	5.5–9.0; 6.5–8.0	IDA
	Carbon sources	starch, yeast extract, lactose, galactose, fructose, lactate, acetate, dextrin	IDA
MIGS-6	Habitat	Marine, hydrothermal sediment	IDA
MIGS-6.3	Salinity	0–5 %	IDA
MIGS-22	Oxygen requirement	Aerobic	IDA
MIGS-15	Biotic relationship	Free-living	IDA
MIGS-14	Pathogenicity	Non-pathogen	NAS
MIGS-4	Geographic location	Troll Wall vent, Arctic Mid-Ocean ridge	IDA
MIGS-5	Sample collection	July 2012	IDA
MIGS-4.1	Latitude	71.29665 N	IDA
MIGS-4.2	Longitude	5.773133 W	IDA
MIGS-4.3	Depth	470m	IDA

Evidence codes – *IDA* Inferred from Direct Assay, *TAS* Traceable Author Statement (i.e., a direct report exists in the literature), *NAS* Non-traceable Author Statement (i.e., not directly observed for the living, isolated sample, but based on a generally accepted property for the species, or anecdotal evidence). These evidence codes are from the Gene Ontology project [63]

The strain produced acid, however no gas production was observed from the following carbohydrate substrates using API 50CH stripes and CHB/E medium (BioMérieux, France): D-fructose, glycerol, esculin, D-maltose, D-saccharose, D-trehalose, D-melezitose, amidon (starch), D-turanose, methyl-αD-glucopyranoside and potassium 5-ketogluconate. Weak acid was produced from D-glucose, D-mannose, methyl-αD-mannopyranoside, N-acetyl-glucosamine, D-lactose, D-melibiose, inulin, D-raffinose, glycogen, xylitol, (gentiobiose), D-lyxose and D-tagatose. In the API Zym panel (BioMérieux, France), strong activity was determined for alkaline phosphatase, esterase (C4), esterase/lipase (C8), leucine arylamidase, α-chymotrypsin, acidic phosphatase and α-glucosidase. Weak activity was observed for lipase (C14), valine arylamidase, cysteine arylamidase, naphtol-AS-BI-phosphohydrolase, β-glucuronidase and β-glucosidase.

*Geobacillus* sp. 12AMOR1 was catalase positive using 3 % hydrogen peroxide. Tests using diatabs (Rosco Diagnostics) identified the isolate as oxidase positive and urease negative.

## Genome sequencing information

### Genome project history

The complete genome sequence and annotation data of *Geobacillus* sp. 12AMOR1 have been deposited in DDBJ/EMBL/GenBank under the accession number CP011832.1. Sequencing was performed at the Norwegian Sequencing Centre in Oslo, Norway [31]. Assembly and finishing steps were performed at the Centre for Geobiology, University of Bergen, Norway. Annotation was performed using the Prokka automatic annotation tool [32] and manually edited to fulfill NCBI standards. Table 2 summarizes the project information and its association with MIGS version 2.0 compliance [33].

**Table 2:** Genome sequencing information

MIGS ID	Property	Term
MIGS-31	Finishing quality	Finished
MIGS-28	Libraries used	Pacific Biosciences 10 kb library
MIGS-29	Sequencing platform	PacBio
MIGS-31.1	Fold coverage	88x
MIGS-30	Assemblers	Hierarchical Genome Assembly Process (HGAP) v2
MIGS-32	Gene calling method	Prodigal
	Locus tag	GARCT, pGARCT
	Genebank ID	Chromosme CP011832
		Plasmid CP011833
	Genebank date of release	June 15, 2015
	BioProject ID	PRJNA277925
	GOLD ID	Gp0115795
MIGS-13	Source Material Identifier	DSM 100439
	Project relevance	Bioprospecting

### Growth conditions and genomic DNA preparation

A pure culture of the isolated *Geobacillus* sp. 12AMOR1 was cultivated on 50 ml LB media for 18 h at 60 °C. After harvesting the cells by centrifugation at 8,000 x g for 10 min high-molecular DNA for sequencing was obtained using a modified method of Marmur [34]. In short: The pellet was suspended in a solution of 1 mg/ml Lysozyme (Sigma 62971) in 10 mM TE buffer (pH 8) and incubated at 37 °C for 15 min. After a Proteinase K treatment (40mg/ml final concentration, Sigma P6556) at 37 °C for 15 min, a final concentration of 1 % SDS was added and the solution was incubated at 60 °C for 5 min until clearance of the solution. A final concentration of 1 M sodium perchlorate (Sigma-Aldrich 410241) was added and the solution well mixed, before an equal volume of Phenol:Chloroform:Isoamylalcohol (25:24:1) was added and the solution gently shaken on a Vortexer for 10 min. After centrifugation at 5,000 x g for 10 min the upper phase was collected and the nucleic acids again extracted twice with Chloroform:Isoamylalcohol (24:1). The nucleic acids was precipitated with 2 volumes of ice cold 100 % ethanol on ice for 60 min, washed in 70 % ethanol, dried and dissolved in 2 ml solution of 50 μg/ml RNase A (R6513 [Sigma]) in TE buffer for RNase treatment at 37 °C for 30 min. One deproteinizing step with Chloroform:Isoamylalcohol was performed as above. A final concentration of 0.3M Sodium Acetate pH 5.2 was added to the DNA solution and the DNA was precipitated using 100 % ethanol as described above. The dried pellet was dissolved in 100 μl 10 mM Tris.HCL (pH = 8) over night at 4°C.

### Genome sequencing and assembly

Approximately 200 μg of genomic DNA was submitted for sequencing. In short, a library was prepared using Pacific Biosciences 10 kb library preparation protocol. Size selection of the final library was performed using BluePippin (Sage Science). The library was sequenced on Pacific Biosciences RS II instrument using P4-C2 chemistry. In total, two SMRT cells were used for sequencing. Raw reads were filtered and *de novo* assembled using SMRT Analysis v. 2.1 and the protocol HGAP v2 (Pacific Biosciences) [35]. The consensus polishing process resulted in a highly accurate self-overlapping contig, as observed using Gepard dotplot [36], with a length of 3,426,502 bp, in addition to a self-overlapping 45,474 bp plasmid. Circularization and trimming was performed using Minimus2 included in the AMOS software package [37]. The circular chromosomal contig and plasmid was polished and consensus corrected twice using the RS_Resequencing protocol in SMRT Analysis v. 2.1. The final polishing resulted in a 3,410,035 bp finished circular chromosome and a 32,689 bp circular plasmid, with a consensus concordance of 99.9 %. The chromosome was manually reoriented to begin at the location of the dnaA gene.

### Genome annotation

The protein-coding, rRNA, and tRNA gene sequences were annotated using Prodigal v. 2.6 [38], RNAmmer v. 1.2 [39] and Aragorn v. 1.2 [40] as implemented in the Prokka automatic annotation tool v. 1.11 [32].

## Genome properties

**Fig. 3:**
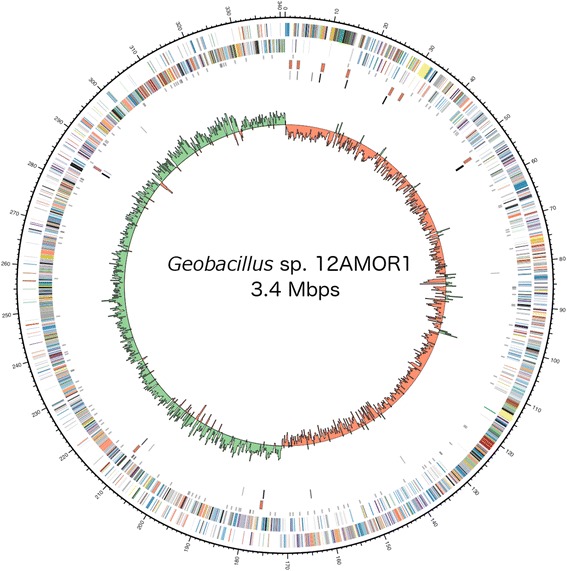
Circular representation of the *Geobacillus* sp. 12AMOR1 draft genome displaying relevant genome features. Circles representing the following (from center to outside): *1*, G + C skew [(G – C)/(G + C) using a 2-kbp sliding window] (green, positive G + C skew; red, negative G + C skew); *2*, tRNAs (black); *3*, rRNA operons (red); *4*, CDS with signal peptides; *5*, Coding DNA sequence (CDS) on the reverse strand; *6*, CDS on the forward strand. Colour coding of CDS was based on COG categories. The figure was build using Circos version. 0.67-6 [54]

The genome of *Geobacillus* sp. 12AMOR1 includes one plasmid of 32,689 bp (47 % G + C content), with one circular chromosome of 3,410,035 bp (52 % G + C content). The main chromosome contained 10 rRNA operons and 88 tRNAs and predicted to encode 3323 protein-coding genes (Table 3 and Fig. 3). 2454 of the protein-coding genes were assigned to a putative function. Identification of peptidases and carbohydrate-degrading enzymes was performed using the MEROPS peptidase database [41] and dbCAN [42], respectively. Using the PHAST web server for the detection of prophages [43], two prophage regions were detected, one intact (56.1Kb: 2476493–2532633) and one incomplete (7.7 Kb: 2811872–2819623). 46 % of the intact prophage protein-coding genes were related to the deep-sea thermophilic bacteriophage GVE2 (NC_009552). The 32.7 Kbps plasmid encoded 34 protein-coding genes.

**Table 3:** Summary of genome: one chromosome and one plasmid

Lable	Size (Mb)	Topology	RefSeq ID
Chromosome	3.4	circular	NZ_CP011832.1
Plasmid	0.32	circular	NZ_CP011833.1

## Insights from the genome sequence

The genome of *Geobacillus* sp. 12AMOR1 encodes for 3323 protein-coding genes (Table 4). Of those proteins 26.15 % could not be annotated towards a specific function and remain hypothetical. In total, 92.66 % of the proteins could be assigned to a COG functional category. The COG functional categories included replication, recombination and repair (9.4 %); amino acid transport and metabolism (6.9 %); inorganic ion transport and metabolism (3.9 %); energy production and conversion (4.17 %); cell wall/membrane/envelop biogenesis (3.7 %) and carbohydrate transport and metabolism (3.8 %) amongst others (Table 5). In the dbCAN analysis, 108 proteins were assigned for one or more functional activities within the CAZy families, which catalyzes the breakdown, biosynthesis or modification of carbohydrates and glycoconjugates [44, 45]. *Geobacillus* sp. 12AMOR1 hydrolyzes starch, dextrin, gelatin, casein and DNA, and utilized sugars such as D-glucose, D-galactose, D-mannose, D-maltose, D-lactose, D-melibiose, D-saccharose, D-trehalose, D-raffinose and glycogen. CDSs encoding for enzymes to metabolize the above mentioned substrates were identified by genome prediction, homology search or mapping onto pathways using the KEGG Automatic Annotation Server [46] server. Furthermore, the isolate was able to grow on the complex carbon polymers xylan, chitin and α-cellulose, however the pathways for such polymer degradation were not identified in the genome. In contrast, pathways for utilization of D-mannitol, arbutin and salicin were identified, although utilization involving acid production was not observed. In comparison with other *Geobacillus* strains, 12AMOR1 harbors less gene modules involved in hydrolysis and utilization of complex carbohydrates [8, 12].

**Table 4:** Statistic of chromosomal genome, including nucleotide content and gene count levels

Attribute	Value	% of total
Genome size (bp)	3,410,035	100.00
DNA coding (bp)	2,936,125	86.1
DNA G + C (bp)	1,775,346	
DNA scaffolds	1	
Total genes	3,441	100.00
rRNA operons	10	
rRNA genes	29	0.83
tRNA genes	88	2.5
tmRNA	1	0.03
Protein coding genes	3,323	95.57
Genes with function prediction	2,454	70.58
Genes assigned to COGs	3,079	88.55
Genes with signal peptids	147	4.23
Genes assigned to prophages	92	2.65
CRISPR repeats	4

**Table 5:** Number of genes associated with general COG functional categories

Code	Value	%age^a^	Description
J	146	4,4	Translation, ribosomal structure and biogenesis
A	0	0	RNA processing and modification
K	153	4,6	Transcription
L	313	9,4	Replication, recombination and repair
B	0	0	Chromatin structure and dynamics
D	31	0,93	Cell cycle control, cell division, chromosome partitioning
V	32	0,96	Defense mechanisms
T	92	2,8	Signal transduction mechanisms
M	123	3,7	Cell wall/membrane/envelope biogenesis
N	41	1,2	Cell motility
U	31	0,93	Intracellular trafficking, secretion, and vesicular transport
O	93	2,8	Posttranslational modification, protein turnover, chaperones
C	136	4,1	Energy production and conversion
G	127	3,8	Carbohydrate transport and metabolism
E	230	6,9	Amino acid transport and metabolism
F	65	1,9	Nucleotide transport and metabolism
H	107	3,2	Coenzyme transport and metabolism
I	80	2,4	Lipid transport and metabolism
P	132	3,9	Inorganic ion transport and metabolism
Q	17	0,5	Secondary metabolites biosynthesis, transport and catabolism
R	0	0	General function prediction only
S	1130	34	Function unknown
-	244	7,3	Not in COGs

^a^the total is based on the number of protein coding genes in the annotated genome

Enzymes involved in protein degradation have been analyzed using MEROPS. In total 126 proteinases were identified. Of those 18 carried a signal peptide identified by SignalP [47] and could be responsible for the extracellular degradation of proteins. *Geobacillus* sp. 12AMOR1 showed strong enzymatic activities for esterase (C4), esterase/lipase (C8), leucine arylamidase, α-chymotrypsin, α-glucosidase, alkaline and acidic phosphatase and weak activity for lipase (C14), valine arylamidase, cysteine arylamidase, β-glucosidase, β-glucuronidase and naphtol-AS-BI-phosphohydrolase.

**Fig. 4:**
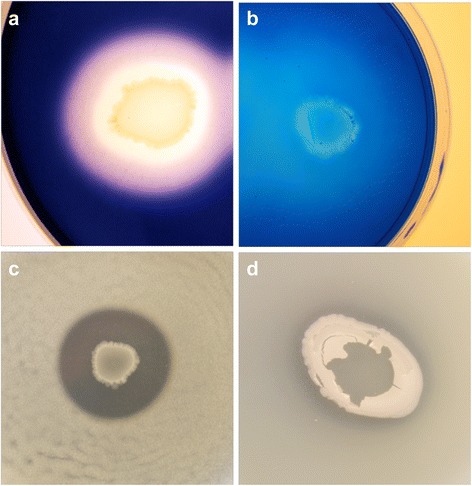
Functional activity screening. Degradation halos around colonies of *Geobacillus* sp. 12AMOR1 growing on agar plates supplemented with **a**, starch; **b**, gelatin; **c**, skim milk and **d**, DNA

The *Geobacillus* sp. 12AMOR1 was screened for the following enzymatic activities; α-amylases, gelatinases, caseinases, lipases, chitinases, xylanases [48–53] and DNase at 60 °C. AG agar plates containing 0.1 % (w/v) yeast extract were used supplemented with 1 % (w/v) starch, 0.5 % (w/v) gelatin, 1 % (w/v) skim milk, 1 % (v/v) olive oil, 1 % (v/v) Tween20, 1 % (v/v) Tween80, 0.5 % (w/v) chitin, 0.5 % (w/v) xylan, respectively. DNase activity was screened on DNase Test Agar (Difco). The strain exhibited hydrolytic enzymatic activity for starch, gelatin, skin milk and DNA (Fig. 4). In addition, growth on plates containing olive oil, chitin and xylan were observed, however no hydrolytic activity could be detected. Putative genes encoding for α-amylase, glycosylase, protease and DNase activity were identified in the genome based on annotation or by homology search (Table 6).

**Table 6:** Candidate genes coding for putative amylase, proteinase and DNase activities identified in *Geobacillus* sp. 12AMOR1 draft genome

Putative gene	Annotation	Size (aa)
Amylase		
GARCT_00588	alpha-amylase	555
GARCT_00679	Neopullulanase	588
GARCT_00683	alpha-amylase	511
GARCT_01758	Trehalose hydrolase	563
GARCT_02913	Glycogen debranching enzyme	680
Glycosylases		
GARCT_00799	Lysozyme	207
GARCT_00912	Dextransucrase	903
GARCT_01278	putative polysaccharide deacetylase PdaA precursor	327
GARCT_01944	Rhamnogalacturonan acetylesterase RhgT	279
GARCT_02324	6-phospho-β-glucosidase	490
GARCT_03212	Putative lysozyme/beta-N- acetylglucosaminidase precursor	1279
GARCT_03220	Putative lysozyme	772
GARCT_03420	Sucrose-6-phosphate hydrolase/GH32_beta_fructosidase	481
Proteases		
GARCT_00241	Serine protease	453
GARCT_00377	Serine protease S01	401
GARCT_00795	Oligoendopeptidase M03	607
GARCT_00799	Peptidase M23	208
GARCT_00975	Oligoendopeptidase M03	564
GARCT_01122	Lon protease	340
GARCT_01527	Serine protease	453
GARCT_01552	Peptidase M32	500
GARCT_01840	Oligoendopeptidase M03	618
GARCT_02099	Aminopeptidase M29	413
GARCT_02390	Dipeptidase M24	353
GARCT_02553	ATP-dependent Clp proteolytic subunit	244
GARCT_02603	Protease	422
GARCT_02604	Peptidase U32	309
GARCT_02662	Peptidase M23	256
GARCT_02693	Lon protease 1	776
GARCT_02694	Lon protease 2	558
GARCT_02769	Aminopeptidase M42	362
GARCT_02850	Aminopeptidase M42	358
GARCT_02860	Putative dipeptidase	471
GARCT_02867	Neutral protease M04	548
GARCT_02978	Aminopeptidase M17	497
GARCT_03009	Peptidase M23	331
GARCT_03106	ATP-dependent Clp proteolytic subunit	197
GARCT_03137	Serine protease S41	480
GARCT_03221	Thermitase	875
GARCT_03224	Stearolysin M4/S8	1338
GARCT_03453	Trypsin-like serine protease	407
DNase		
GARCT_00042	Putative Ribonuclease YcfH	257
GARCT_00112	Ribonuclease III C	141
GARCT_00224	Putative deoxyribonuclease YcfH	251
GARCT_00623	3’-5’ exoribonuclease YhaM	326
GARCT_00659	Nuclease SbcCd subunitD	395
GARCT_01396	Restriction endonuclease	354
GARCT_01547	Putative Exonuclease (hypothetical protein)	421
GARCT_01867	Extracellular ribonuclease	309
GARCT_02076	HNH endonuclease	420
GARCT_02373	Exodeoxyribonuclease VII, small subunit	77
GARCT_02374	Exodeoxyribonuclease VII, large subunit	449
GARCT_02456	Putative endonuclease 4	300
GARCT_02557	HNH Endonuclease	165
GARCT_02575	HNH Endonuclease	184
GARCT_02948	Endonuclease YokF	303
GARCT_03029	Endonuclease YhcR	461

**Fig. 5:**
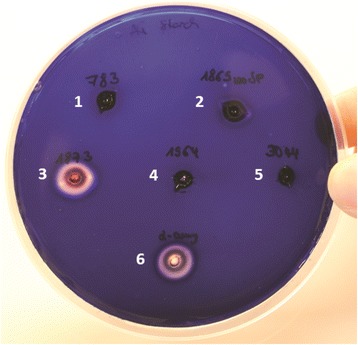
Activity of purified *Geobacillus* sp. 12AMOR1 alpha-amylases on starch agar plates. As the plate was colored with iodine solution degradation appear as clear zones. 1) Trehalose hydrolase (GARCT_01758), 2) Alpha-amylase (GARCT_00683), 3) Neopullulanase (GARCT_00679), 4) Alpha-amylase (GARCT_00588), 5) Glycogen debranching enzyme (GARCT_02913), 6) Alpha-amylase from *B. licheniformis* (Sigma-Aldrich)

Due to their broad biotechnological applications, such as in food processing, detergents or bioethanol production [17], identifying novel α-amylases is still of biotechnological interest. Five genes encoding for α-amylases of the GH13 family (Table 6) were identified by dbCAN analysis. The neopullulanase (GARCT_00679; AKM17981) was cloned using following primers F: AGG AGA TAT ACC ATG CAA AAA GAA GCC ATT CAC CAC CGC, R: GTG ATG GTG ATG TTT CCA GCT TTC AAC TTT ATA GAG CAC AAA CCC, and expressed in *E. coli* BL21 (DE3). The protein GARCT_00679 was purified in high amounts from *E. coli* and revealed a melting temperature of 76.4 °C in differential scanning calorimetry (DSC) analysis. As expected this value was elevated from the optimal growth temperature of the isolate. Using purified protein solution on 1 % starch-agar plates only GARCT_00679 showed starch degradation capacity comparable with the reference alpha amylase from *B. licheniformis* (Sigma-Aldrich) (Fig. 5).

## Conclusions

The starch degrading, thermophilic *Geobacillus* sp. 12AMOR1, isolated from an Arctic deep-sea hydrothermal vent system, revealed a 3.4 Mbp complete genome composed of a circular chromosome and a plasmid. The genome and plasmid have been deposited at GenBank under the accession numbers CP011832 and CP011833, respectively. The genome size within the genus ranges between 3.35 and 3.84 Mbp (RefSeq: NZ_BATY00000000.1; NC_014650.1), therefore *Geobacillus.* sp. 12AMOR1 belongs with 3.4 Mbp to the smaller genomes. The G + C content of 52 % is within the average of the genus.

16S rRNA analysis identified the isolate belonging to *Geobacillus stearothermophilus*, whereas DDH analysis with 13 *Geobacillus* genomes indicated a slightly distant relationship towards the other *Geobacillus* strains. In the phylogenetic analysis *Geobacillus* sp. 12AMOR1 was located in a sub-cluster apart from the type strain *G. stearothermophilus*DSM 22^T^ within in the same cluster.

When comparing the phenotypical characteristics of diverse *G. stearothermophilus* strains in the literature, the profile varies from strain to strain [1, 14, 29]. Most of the phenotypical features of *Geobacillus* sp*.* 12AMOR1 lie within those variations. Minor divergences of 12AMOR1 are acid production from potassium 5-ketogluconate and lactose (and maybe gentiobiose), utilization of lactose, and being oxidase positive. Those phenotypical characteristics are not sufficient to support a differentiation between *G. stearothermophilus* and *Geobacillus* sp. 12AMOR1, even though the DDH analysis suggests a distant relationship.

Although *Geobacillus* sp. 12AMOR1 features less genes encoding for carbohydrate degrading enzymes in comparison with other *Geobacillus* strains, a multiplicity of interesting enzymes, applicable for biotechnology, was identified by genome annotation and by activity screening. Hence, *Geobacillus* sp. 12AMOR1 can serve as a source of functional enzymes for future bioprospecting.

## Additional files

Additional file 1:
**16S rRNA sequence identities towards** ***Geobacillus sp.*** **12AMOR1.** Chosen blast hits with the highest sequence identity (98 %) towards the preliminary partial 16S rRNA gene of *Geobacillus* sp. strain 12AMOR1 using the megablast algorithm a standalone blastn [23] against 16S ribosomal RNA (Bacteria and Archaea database). (DOCX 14 kb) Additional file 2:
**Digital DNA-DNA Hybridization of Geobacillus sp.12AMOR1 genome towards other Geobacillus genomes performed by the** **Genome-to-Genome** **Distance Calculator (GGDC) 2.0 using formula 2 (identities/HSP length).** The genomes of *G. kaustophilus* HTA426 [NC_006510.1], *G. stearothermophilus* strain X1 [CP008855.1], *G. stearothermophilus* NUB3621 isolate 9A5 [CM002692.1], *G. thermoleovorans* CCB_US3_UF5 [CP003125.1], *G. thermodenitrificans* NG80-2 [NC_009328.1], *G. vulcani* PSS1 [gb|JPOI01000001.1], *Geobacillus* sp. C56-T3 [NC_014206.1], *Geobacillus* sp. GHH01 [CP004008.1], *Geobacillus* sp. JF8 [CP006254.2], *Geobacillus* sp. WCH70 [CP001638.1], *Geobacillus* sp. Y4.1MC1 [NC_014650.1], *Geobacillus* sp. Y412MC52 [NC_014915.1], *Geobacillus* sp. Y412MC61 [NC_013411.1] was used for comparison. The genome of *Bacillus licheniformis* ATCC 14580^T^ [NC_006270.3] was used as outgroup. (PDF 67 kb)

## Abbreviations

CAZy: Carbohydrate active enzyme
GH13: Glycoside hydrolase group 13
AG: *Archaeoglobus* medium
