# Supplementary material for: Isolation and complete genome sequence of the thermophilic Geobacillus sp. 12AMOR1 from an Arctic deep-sea hydrothermal vent site
Source: Stand Genomic Sci. 2016 Feb 24;11:16. doi: 10.1186/s40793-016-0137-y (PMC4765119; doi:10.1186/s40793-016-0137-y)
Supplement: Additional file 1: — 16S rRNA sequence identities towards Geobacillus sp. 12AMOR1. Chosen blast hits with the highest sequence identity (98 %) towards the preliminary partial 16S rRNA gene of Geobacillus sp. strain 12AMOR1 using the megablast algorithm a standalone blastn [23] against 16S ribosomal RNA (Bacteria and Archaea database). (DOCX 14 kb) [file 40793_2016_137_MOESM1_ESM.docx]

| **Description** | **Query cover %** | **E-value** | **Ident %** | **Acceccion** |
| --- | --- | --- | --- | --- |
| *Geobacillus stearothermophilus* strain DSM 22 16S ribosomal RNA gene, complete sequence | 98 | 0.0 | 98 | NR_114762.1 |
| *Geobacillus stearothermophilus* strain R-35646 16S ribosomal RNA gene, partial sequence | 98 | 0.0 | 98 | NR_116987.1 |
| *Geobacillus subterraneus* strain 34 16S ribosomal RNA gene, partial sequence | 98 | 0.0 | 98 | NR_025109.1 |
| *Geobacillus zalihae* strain NBRC 101842 16S ribosomal RNA gene, partial sequence | 98 | 0.0 | 98 | NR_114014.1 |
| *Geobacillus thermoleovorans* strain BGSC 96A1 16S ribosomal RNA gene, complete sequence | 98 | 0.0 | 98 | NR_115286.1 |
| *Geobacillus thermocatenulatus* strain BGSC 93A1 16S ribosomal RNA gene, complete sequence | 98 | 0.0 | 98 | NR_043020.1 |
| *Geobacillus vulcani* strain 3S-1 16S ribosomal RNA gene, partial sequence | 98 | 0.0 | 98 | NR_025426.1 |
| *Geobacillus kaustophilus* strain BGSC 90A1 16S ribosomal RNA gene, complete sequence | 98 | 0.0 | 98 | NR_115285.1 |
