# Supplementary material for: Isolation and complete genome sequence of the thermophilic Geobacillus sp. 12AMOR1 from an Arctic deep-sea hydrothermal vent site
Source: Stand Genomic Sci. 2016 Feb 24;11:16. doi: 10.1186/s40793-016-0137-y (PMC4765119; doi:10.1186/s40793-016-0137-y)
Supplement: Additional file 2: — Digital DNA-DNA Hybridization of Geobacillus sp.12AMOR1 genome towards other Geobacillus genomes performed by the Genome-to-Genome Distance Calculator (GGDC) 2.0 using formula 2 (identities/HSP length). The genomes of G. kaustophilus HTA426 [NC_006510.1], G. stearothermophilus strain X1 [CP008855.1], G. stearothermophilus NUB3621 isolate 9A5 [CM002692.1], G. thermoleovorans CCB_US3_UF5 [CP003125.1], G. thermodenitrificans NG80-2 [NC_009328.1], G. vulcani PSS1 [gb|JPOI01000001.1], Geobacillus sp. C56-T3 [NC_014206.1], Geobacillus sp. GHH01 [CP004008.1], Geobacillus sp. JF8 [CP006254.2], Geobacillus sp. WCH70 [CP001638.1], Geobacillus sp. Y4.1MC1 [NC_014650.1], Geobacillus sp. Y412MC52 [NC_014915.1], Geobacillus sp. Y412MC61 [NC_013411.1] was used for comparison. The genome of Bacillus licheniformis ATCC 14580T [NC_006270.3] was used as outgroup. (PDF 67 kb) [file 40793_2016_137_MOESM2_ESM.pdf]

Species	1.	2.	3.	4.	5.	6.	7.	8.	9.	10.	11.	12.	13.	14.	15.
1. *Geobacillus* sp. 12AMOR1	**100**														
2. *G. kaustophilus* HTA426	39.80	**100**													
3. *G. stearothermophilus* strain X1	41.50	28.10	**100**												
4. *G. stearothermophilus* NUB3621 isolate 9A5	21.50	21.10	28.10	**100**											
5. *G. thermoleovorans* CCB_US3_UF5	40.50	82.30	27.70	20.90	**100**										
6. *G. thermodenitrificans* NG80-2	27.50	27.30	41.80	21.50	27.50	**100**									
7. *G. vulcani* PSS1 N685DRAFT	31.50	44.00	36.40	20.70	43.80	27.20	**100**								
8. *Geobacillus* sp. C56-T3	37.80	70.30	37.70	20.30	68.50	27.10	42.90	**100**							
9. *Geobacillus* sp. GHH01	41.50	67.40	28.40	21.10	67.50	27.10	41.60	68.80	**100**						
10. *Geobacillus* sp. JF8	33.00	29.50	28.50	20.30	29.50	29.60	27.90	29.30	30.40	**100**					
11. *Geobacillus* sp. WCH70	30.80	28.80	27.60	29.00	29.30	23.30	22.90	25.30	27.90	23.30	**100**				
12. *Geobacillus* sp. Y4.1MC1	22.10	22.20	29.90	26.70	21.70	22.20	21.40	20.40	21.90	20.70	31.30	**100**			
13. *Geobacillus* sp. Y412MC52	37.40	70.00	37.90	20.30	68.90	26.90	42.90	83.59	67.80	29.40	25.80	20.30	**100**		
14. *Geobacillus* sp. Y412MC61	37.30	70.00	37.90	20.30	68.90	26.90	42.90	83.50	67.80	29.40	25.70	20.30	100	**100**	
15. *Bacillus licheniformis* strain ATCC 14580	34.70	30.70	32.50	30.80	31.00	32.40	31.00	30.40	29.70	33.90	28.30	28.40	30.40	30.40	**100**
